# Comparative sequence analysis of nitrogen fixation-related genes in six legumes

**DOI:** 10.3389/fpls.2013.00300

**Published:** 2013-08-22

**Authors:** Dong Hyun Kim, Swathi Parupalli, Sarwar Azam, Suk-Ha Lee, Rajeev K. Varshney

**Affiliations:** ^1^Center of Excellence in Genomics, International Crops Research Institute for the Semi-Arid Tropics (ICRISAT)Hyderabad, India; ^2^Department of Plant Science, Seoul National UniversitySeoul, South Korea; ^3^Research Institute for Agriculture and Life Sciences, Seoul National UniversitySeoul, South Korea; ^4^CGIAR Generation Challenge Programme, c/o CIMMYTMexico DF, Mexico

**Keywords:** nitrogen fixation, legume, comparative analysis, Ks, evolution

## Abstract

Legumes play an important role as food and forage crops in international agriculture especially in developing countries. Legumes have a unique biological process called nitrogen fixation (NF) by which they convert atmospheric nitrogen to ammonia. Although legume genomes have undergone polyploidization, duplication and divergence, NF-related genes, because of their essential functional role for legumes, might have remained conserved. To understand the relationship of divergence and evolutionary processes in legumes, this study analyzes orthologs and paralogs for selected 20 NF-related genes by using comparative genomic approaches in six legumes i.e., *Medicago truncatula* (*Mt*), *Cicer arietinum, Lotus japonicus, Cajanus cajan* (*Cc*), *Phaseolus vulgaris* (*Pv*), and *Glycine max* (*Gm*). Subsequently, sequence distances, numbers of synonymous substitutions per synonymous site (Ks) and non-synonymous substitutions per non-synonymous site (Ka) between orthologs and paralogs were calculated and compared across legumes. These analyses suggest the closest relationship between *Gm* and *Cc* and the highest distance between *Mt* and *Pv* in six legumes. Ks proportional plots clearly showed ancient genome duplication in all legumes, whole genome duplication event in *Gm* and also speciation pattern in different legumes. This study also reports some interesting observations e.g., no peak at Ks 0.4 in *Gm*-*Gm*, location of two independent genes next to each other in *Mt* and low Ks values for outparalogs for three genes as compared to other 12 genes. In summary, this study underlines the importance of NF-related genes and provides important insights in genome organization and evolutionary aspects of six legume species analyzed.

## Introduction

Legume is an important class of plants that provides protein in diet for a significant proportion of human population as well as supplies nitrogen to environments. Legumes perform a special symbiotic process called nitrogen fixation (NF) that can fix atmospheric nitrogen (N_2_) to ammonia (NH_3_) by rhizobium. Papilionoideae subfamily contains majority of commercially important legumes as well as model legume species. Papilionoideae subfamily can be divided into two groups. One is Hologalegina (cool season legumes), including *Medicago truncatula* (*Mt*), chickpea (*Cicer arietinum, Ca*), and *Lotus japonicus* (*Lj*), the other is Phaseoloid (warm season legumes), including soybean (*Glycine max, Gm*), common bean (*Phaseolus vulgaris, Pv*), and pigeonpea (*Cajanus cajan, Cc*). In context of understanding biological process of NF, many mutants were developed or identified and NF-related genes were isolated from two model legumes, *Mt* and *Lj* (Kouchi et al., 2010). While genome sequencing projects were initiated earlier in *Mt* (Young et al., 2011) and *Lj* (Sato et al., 2008), genome sequences have become available for crop legumes like soybean (Schmutz et al., 2010), pigeonpea (Varshney et al., 2012), chickpea (Varshney et al., 2013), common bean (http://phytozome.net). Nevertheless, even before the availability of genome sequences, researchers exploited the BAC sequences to understand not only comparative evolutionary history of a range of genes but also genome duplication and divergence events (Schlueter et al., 2007; Shin et al., 2008; Kim et al., 2009). As genome sequences of several legumes have become available in recent years, analysis for speciation and rearrangements is possible in more species.

NF-related genes are very specific and essential to legumes therefore they can be good genomic tools for understanding the process of evolution in legumes. Moreover, morphological differences of nodulation have been used as one of the taxonomic criteria so it is plausible to utilize sequences of NF-related genes for phylogenetic analysis (Sprent, 2000, 2007). After several times of major and minor rearrangements, legumes were diverged into different species (Lavin et al., 2005). Orthologs originate from speciation while paralogs are caused by duplication (Koonin, 2005). It is also important to note that while some genes can be duplicated before speciation and some after speciation. To avoid confusion of such genes with orthologs and paralogs, duplicated genes before speciation are called outparalogs and after speciation are called inparalogs (Koonin, 2005).

Relative timing of duplication of two homologs for a given gene between two species can be estimated by numbers of synonymous substitutions per synonymous site (Ks) value (Koch et al., 2000; Blanc and Wolfe, 2004; Shoemaker et al., 2006). Lower Ks value suggests that divergence between these homologs happened recently. The ratio of number of non-synonymous substitutions per non-synonymous site (Ka) and Ks (Ka/Ks) provides information of the selection pressure in sequence evolution (Hurst, 2002).

In view of the above, this article presents analysis and critical appraisal on 20 NF-related genes for understanding gene-level evolution in six legume species (*Ca, Cc, Gm, Lj, Mt*, and *Pv*) by using comparative genomics approaches.

## Materials and methods

### Gene compilation and sources of sequence data

A list of 52 NF-related genes was utilized from *Gm* genome sequence data (Schmutz et al., 2010). Gene names were taken from gene cloning publications of *Mt* or *Gm* and gene sequences were downloaded from NCBI website. Coding DNA sequences (CDS) of six legumes were downloaded for finding homologs by BLAST from Phytozome [http://phytozome.net] for *Mt* (v3.0), *Pv* (v1.0), *Gm* (v1.1), International Chickpea Genetics and Genome Sequencing Consortium [http://www.icrisat.org/gt-bt/ICGGC/GenomeSequencing.htm] for *Ca* (v1.0), Kazusa DNA Research Institute [http://www.kazusa.or.jp] for *Lj* (v2.5) and International Initiative for Pigeonpea Genomics [http://www.icrisat.org/gt-bt/iipg/genomedata.zip] for *Cc* (v5.0).

### Sequence analysis

Standalone BLAST package, ncbi-blast-2.2.25+ from NCBI was used for homologs search analysis. All NF-related genes were compared against all six legume's CDS using BLASTN program. Further, homology hits were filtered on criterion of 70 % identity and *e*-value cut-off of ≤1E−50 using in-house perl script. All potential hits or homologous sequences were extracted from CDS databases of each legume. Finally, genes were selected by using a criteria of presence of homologs in at least four out of six legumes species (Table 1). All NF-related genes were clustered into gene families using orthoMCL v1.4 (Li et al., 2003). Bidirectional best hits by BLAST was used for confirmation of orthologs in six species (Zhang and Leong, 2010).

**Table 1:** **List of 20 NF-related genes analyzed in six legume species**.

Gene name^*^	***Medicago truncatula***	***Cicer arietinum***	Lotus japonicus^**^	***Cajanus cajan***	***Phaseolus vulgaris***	Glycine max^***^
*MtDMI1*^1^	Medtr2g005620	Ca_00033	Lj6.CM0508.260.r2.m	C.cajan_17266	Phvulv091019046m	Glyma12g28860
				C.cajan_11017		Glyma16g00500
						Glyma19g45310
*MtDMI2*^2^	Medtr5g032400	Ca_11537	Lj2.CM0177.340.r2.m	C.cajan_12295	Phvulv091027352m	Glyma09g33510
		Ca_17066				Glyma01g02460
*MtDMI3*^3^	Medtr8g047760	Ca_15707	Lj3.LjT02O17.60.r2.m	C.cajan_46131	Phvulv091013422m	Glyma15g35070
	Medtr5g009940					Glyma08g24360
						Glyma10g11020
*MtERN1*^4^	Medtr7g102550	Ca_08232	Lj1.CM0104.2670.r2.m	C.cajan_08385	Phvulv091004951m	Glyma16g04410
	Medtr6g031080			C.cajan_16144		Glyma19g29000
*MtERN3*^5^	Medtr6g015110	Ca_08582	Lj4.CM0046.750.r2.a	C.cajan_23330	Phvulv091030938m	Glyma08g12130
	Medtr4g134350					Glyma05g29011
*MtFLOT2*^6^	Medtr3g137870			C.cajan_09162	Phvulv091009868m	Glyma06g06930
	Medtr1g099720					Glyma04g06830
*MtIPD3*^7^	Medtr5g027010	Ca_10616	Lj2.CM0803.150.r2.m	C.cajan_12408	Phvulv091016359m	Glyma01g35255
						Glyma09g34695
*MtLIN*^8^	Medtr1g112060	Ca_08341	Lj5.CM0909.400.r2.m	C.cajan_22455	Phvulv091027173m	Glyma10g33851
						Glyma12g29771
*MtLYK3*^9^	Medtr5g093450	Ca_10278	Lj2.CM0545.250.r2.m	C.cajan_09999	Phvulv091021871m	Glyma14g05060
	Medtr5g093440		Lj6.CM0041.460.r2.a	C.cajan_15801		Glyma02g43860
	Medtr5g093730					Glyma02g43850
	Medtr5g093410					
*MtLYR3*^10^	Medtr5g019000	Ca_02085	Lj2.CM0323.420.r2.d	C.cajan_12623	Phvulv091008254m	Glyma11g06750
						Glyma01g38550
						Glyma02g06700
*MtNFP*^10^	Medtr5g018990	Ca_02086	Lj2.CM0323.400.r2.d	C.cajan_12621	Phvulv091008306m	Glyma11g06740
	Medtr8g093910	Ca_16029				Glyma01g38560
*MtNIN*^11^	Medtr5g106690	Ca_09832	Lj2.CM0102.250.r2.m	C.cajan_33924	Phvulv091031090m	Glyma06g00240
				C.cajan_37712	Phvulv091004689m	Glyma04g00210
						Glyma02g48080
*MtNRT1*^12^	Medtr5g093170	Ca_10291	Lj2.CM0826.350.r2.m	C.cajan_09986	Phvulv091021785m	Glyma02g43740
			Lj2.CM0826.370.r2.m			Glyma14g05170
			Lj2.CM0545.330.r2.m			
*MtNSP1*^13^	Medtr8g025000	Ca_10004	Lj3.CM0416.1260.r2.d	C.cajan_27701	Phvulv091018505m	Glyma07g04430
	Medtr5g015580				Phvulv091030806m	Glyma16g01020
	Medtr8g101580				Phvulv091007340m	Glyma05g22460
*MtNSP2*^13^	Medtr3g097800	Ca_26279	Lj1.CM1976.90.r2.m	C.cajan_01355	Phvulv091012665m	Glyma04g43090
	Medtr5g065380	Ca_23494		C.cajan_32376		Glyma06g11610
						Glyma13g02840
*MtRRP1*^14^	Medtr1g074280	Ca_26056	Lj5.CM1077.650.r2.m	C.cajan_33337	Phvulv091005582m	Glyma13g21080
		Ca_19055				Glyma10g07190
*MtSKL1*^15^	Medtr7g121800	Ca_12043	Lj1.CM0012.1100.r2.m	C.cajan_45110	Phvulv091008769m	Glyma03g33850
						Glyma13g20810
						Glyma10g06610
*MtSUNN*^16^	Medtr4g096420	Ca_15399	Lj3.CM0091.1690.r2.m	C.cajan_21258	Phvulv091015304m	Glyma12g04390
	Medtr4g096400	Ca_09375		C.cajan_24880		Glyma11g12186
				C.cajan_39327		
*GmN56*^17^	Medtr1g146810	Ca_13985	Lj5.CM0492.390.r2.m	C.cajan_07899	Phvulv091005854m	Glyma10g44180
		Ca_26114	Lj1.CM0001.650.r2.m	C.cajan_37827		Glyma20g38950
			Lj1.CM0001.690.r2.m	C.cajan_46126		Glyma13g12484
			Lj1.CM0001.710.r2.m			Glyma19g29920
						Glyma19g29880
*GmENOD93*^18^	Medtr8g119590	Ca_06646		C.cajan_46055	Phvulv091017136m	Glyma06g24760
				C.cajan_26197		Glyma05g08400
				C.cajan_26199		Glyma17g12600
						Glyma17g12610
						Glyma05g08380

Orthologs by bidirectional best hit and OrthoMCL were mentioned on the top of the genes listed for each species except GmENOD93 (Medtr8g119590 and Ca_06646).

* Gene names as per the research articles in which these genes were cloned and published.

** Gene name of Lj were changed from chr to Lj for convenience.

*** Underlined genes of Gm were present in syntenic regions.

1 (Ané et al., 2004),

2 (Endre et al., 2002),

3 (Lévy et al., 2004),

4 (Middleton et al., 2007),

5 (Andriankaja et al., 2007),

6 (Haney and Long, 2010),

7 (Messinese et al., 2007),

8 (Kiss et al., 2009),

9 (Smit et al., 2007),

10 (Arrighi et al., 2006),

11 (Marsh et al., 2007),

12 (Morère-Le Paven et al., 2011),

13 (Hirsch et al., 2009),

14 (Arrighi et al., 2008),

15 (Penmetsa et al., 2008),

16 (Elise et al., 2005),

17 (Kouchi and Hata, 1995),

18 (Kouchi and Hata, 1993).

All selected homologous genes were subjected for multiple sequence alignment using Clustal 2 (http://www.clustal.org/clustal2) with default parameter. Phylogenetic trees were constructed by MEGA5 using the neighbor-joining, maximum-likelihood, and maximum-parsimony method with 1000 replicates in the bootstrap test (Tamura et al., 2011). All positions containing gaps and missing sequences were not calculated. Sequence distance, Ks and Ka were calculated between all gene pairs in each NF-related gene by MEGA5. Sequence distance which indicates the extent of similarity between homologs (including orthologs and paralogs) was calculated by the number of base substitutions per site.

## Results and discussion

### Organization of selected nitrogen fixation-related genes

**Figure 1:**
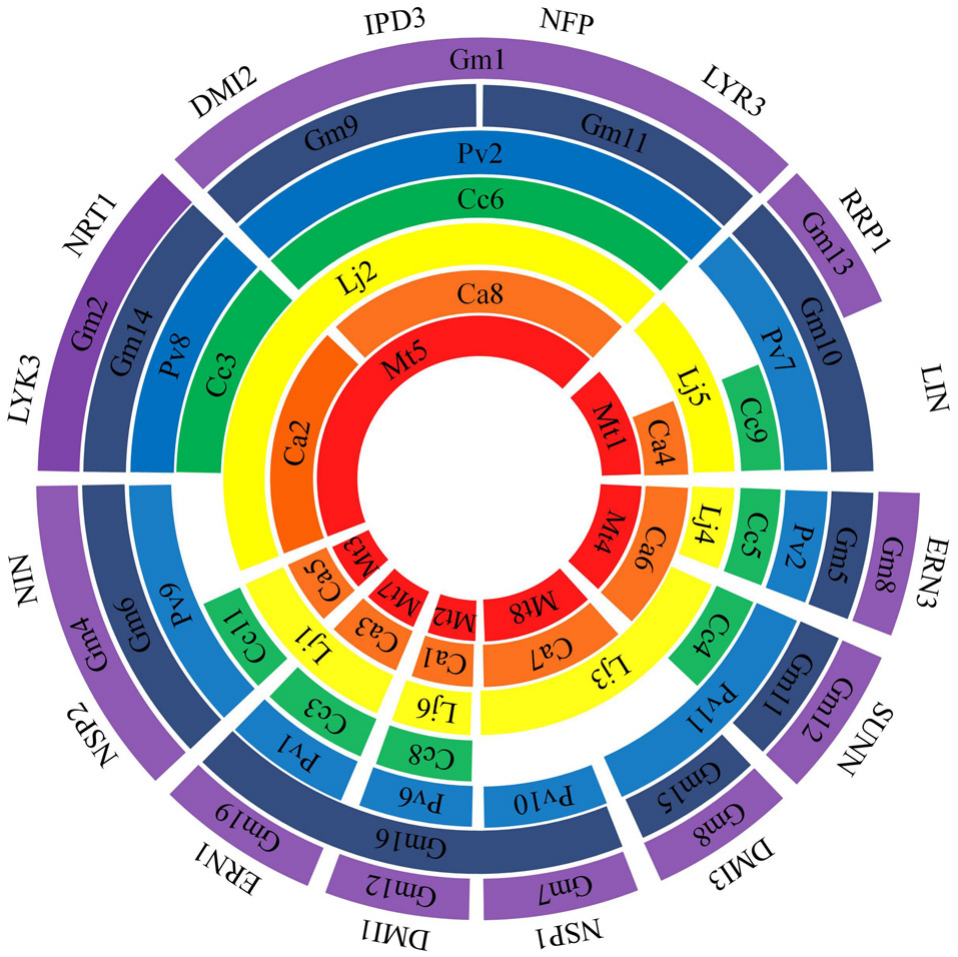
**Comparative genome location of 16 genes in six legume species**. Chromosome/pseudomolecule/linkage group have been shown by arc. Legume species have shown in different colors: *Mt*-red, *Ca*-orange, *Lj*-yellow, *Cc*-green, *Pv*-blue, and *Gm*-dark blue, purple. Gap indicates that the gene is on unmapped scaffold.

Genes responsible for signal pathway and nodulation were also included in the list of NF-related genes in the broad concept of NF pathway. Although many NF-related genes were cloned using traditional genetics (e.g., map-based cloning and forward genetics) approaches in *Mt* and *Lj*, soybean genome sequencing provided occurrence of 52 NF-related genes. All these 52 genes were searched for homology in the genome sequences of *Mt, Lj, Ca, Pv*, and *Cc*. By searching for the presence of orthologs in at least four of six legume species surveyed, a total of 20 NF-related genes were selected for further analysis (Table 1). Orthologs relationships in six legumes were confirmed by bidirectional best hit and orthoMCL. Due to the recent duplication of *Gm* genome, it has two or more homologs (Schmutz et al., 2010). As nomenclature for majority of NF-related genes were given in cloning studies in *Mt* and *Lj*, identified orthologs in the legume genomes surveyed were named accordingly. All 20 NF-related genes were used for placing them on chromosomes/pseudomolecules based on sequence analysis across all the legume crops (Figure 1). For instance, in the case of *Mt* genome, there are seven genes (*DMI2, IPD3, LYK3, LYR3, NFP, NIN*, and *NRT1*) located on *Mt*5. Although *MtNFP* and *MtLYR3* genes were present next to each other, these genes have their own orthologs in different species. As expected because of two times of genome duplication in *Gm*, all genes had at least two paralogs in the genome and most of them were present in the syntenic regions and 18 genes of 20 (except *LIN* and *FLOT2*) had inparalogs. In the case of *DMI3* ortholog in *Gm*, it has a paralog but not present in the syntenic region. Therefore, it is possible that one copy of *LIN* and *FLOT2* could have been deleted and a paralog of *DMI3* might have been relocated after the recent duplication in *Gm* genome.

### Overall sequence distances and synonymous substitution rates

Sequence distance, Ks as well as Ka values were calculated for all possible 922 homolog pairs of 20 NF-related genes in legume genomes surveyed. Comparison of these data showed the lowest sequence distance for 15 out 20 (75%) NF-related genes in *Gm*-*Gm* and 14 (70%) genes showed the lowest Ks in *Gm*-*Gm* (Supplementary Table 1). The lowest sequence distance and Ks resulted from recently duplicated genes in *Gm*. After excluding *Gm*-*Gm* (paralog) relationship, *Gm*-*Cc* orthologs for seven genes have the lowest sequence distance and eight genes have the lowest Ks. On the contrary, *Mt*-*Pv* orthologs for six genes have the highest sequence distance and Ks. These observations imply that *Gm*-*Cc* could be the closest and *Mt*-*Pv* would be the farthest in evolution of six species. To check the evolutionary distance from *Gm* to the other five species, Ks medians (excluding Ks from tandem repeats) were compared (Supplementary Table 2). *Gm*-*Gm* (0.181) has the least Ks median, *Gm*-*Cc* (0.212) and *Gm*-*Pv* (0.212) have same Ks median, *Gm*-*Lj* (0.331) and *Gm*-*Ca* (0.336) have almost similar Ks median and *Gm*-*Mt* (0.398) has the highest Ks median. Based on Ks median values, both *Pv* and *Cc* are the closest to *Gm* followed by *Lj* and *Ca*, and *Mt* is the farthest from *Gm*. While analyzing the evolutionary distances of different species from *Mt, Mt- Ca* has the lowest Ks median (0.282), and higher Ks median was observed for *Mt*-*Cc* (0.405) and *Mt*-*Pv* (0.400). Therefore, by considering only Ks median values, it is not possible to infer the farthest species from *Mt* in the legume evolution. In summary, by considering the maximum number of genes with least Ks and the lowest Ks median across the genes for all orthologs of 20 NF-related genes, *Gm*-*Cc* were found to be the closest followed by *Gm*-*Pv* and *Mt-Pv* were found to be the farthest.

It is well known that Ka is smaller than Ks in natural evolution because of conservation of functional coding genes, therefore non-synonymous change was less frequent in mutation of nucleotides during evolution (Hurst, 2002; Nekrutenko et al., 2002). Average Ka/Ks across 20 NF-related genes in six legumes is 0.69 (Supplementary Table 2). The lower Ka/Ks value (<1) in NF-related genes suggested that most of the genes have remained under negative selection in the course of evolution (Suzuki and Gojobori, 1999). Higher Ka/Ks value (>1) was observed only for *ENOD93* (1.461) but this also does not represent a strong positive selection. Interestingly, about 50% (9 genes) of NF-related genes such as *DMI3* (0.245), *FLOT2* (0.193), *NRT1* (0.255) had very low (<<1) Ka/Ks values which showed a very strong negative selection pressure in course of evolution of legume species. As earlier studies indicated that genes with essential functions have lower Ka/Ks values (Lam et al., 2010; Xu et al., 2012), this study once again underlines the importance and essentiality of the NF-related genes for legume species. Because of this reason, these genes have remained more conserved in speciation and rearrangements during the evolution of legume species.

**Figure 2:**
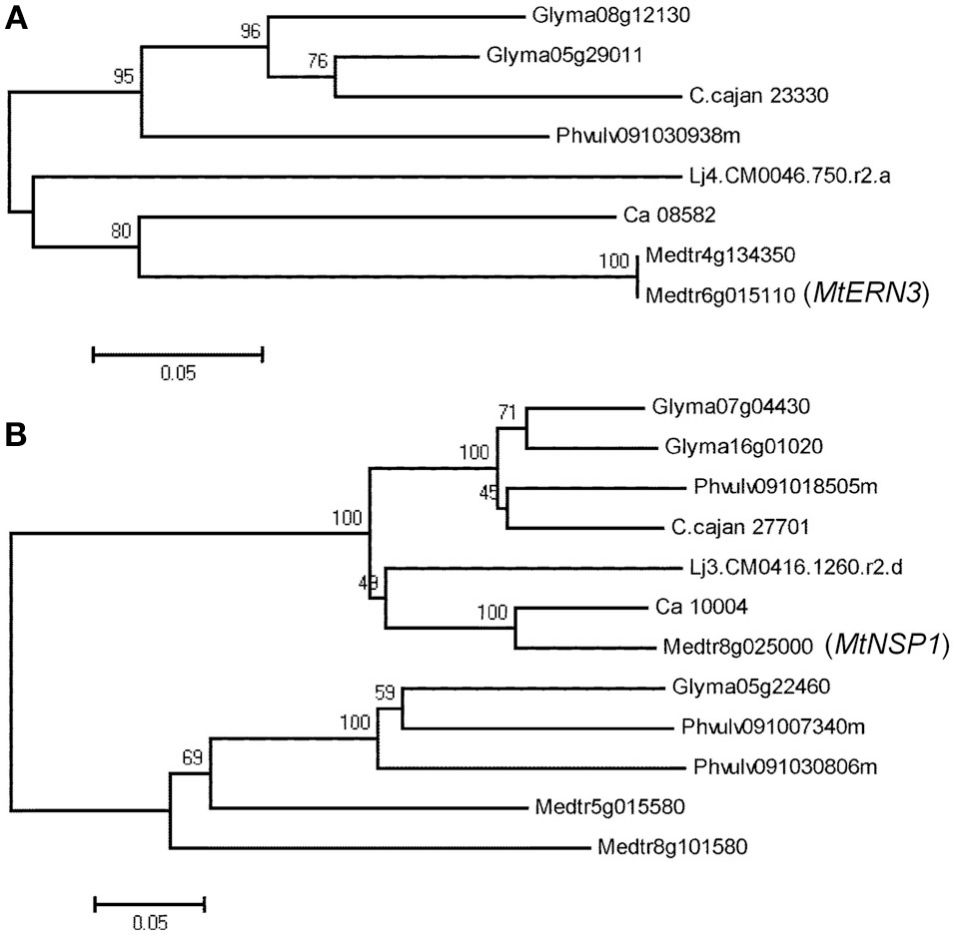
**Phylogenetic trees based on sequence data using the neighbor-joining method**. The percentage of replicate trees in which the associated taxa clustered together in the bootstrap test (1000 replicates) are shown above the branches **(A)**. *ERN3* has one cluster. **(B)**. *NSP1* has two clusters.

Phylogenetic relationship analysis with neighbor-joining method based on sequence diversity for all analyzed genes in the six legume species showed two types of phylogenetic trees. For instance, in the case of five genes (*DMI2, ERN3, IPD3, NRT1* and *RRP1*), only one cluster (clade) was observed (Figure 2A, Supplementary Figure 1). In the remaining 15 genes, phylogenetic trees consist of two clusters (Figure 2B). Genes belonging to the first type of phylogenetic trees, that have only one cluster (and not have outparalogs), can be considered to be originated from the single gene of a common ancestor. On the other hand, genes belonging to the second type of phylogenetic trees, that consist of two clusters, may be considered to be duplicated before speciation (Koonin, 2005). While analyzing two types of clusters for all the genes, average sequence distances within and between clusters were observed as 0.237 and 0.437, respectively. Similarly, Ks values within and between clusters were 0.290 and 0.510, respectively. This Ks value (0.510) supports that genes belonging to different clusters could be originated from the different genes of the common ancestor before speciation, because Ks peaks were observed at Ks 0.4 (Koonin, 2005). In addition to neighbor-joining method, maximum-likelihood and maximum-parsimony methods were also used for phylogenetic analysis (Supplementary Figures 2, 3). Most of the cases displayed similar trees compared with neighbor-joining trees except *LIN, LYK3*, and *LYR3*. These three genes had one or two genes in different cluster. OrthoMCL and bidirectional best hit were also used for confirmation of orthologs and they showed same results (Supplementary Table 3). It is an interesting observation that *ENOD93* had two different orthologs in *Gm* and *Cc* by OrthoMCL and it was identical to the phylogenetic tree of *ENOD93* which had only three genes in the first cluster (Supplementary Figure 1). Glyma06g24760, Phvulv091017136m and C.cajan_46055 are orthologs and the other genes could be outparalogs to them.

### Elucidation of duplication and divergence

In general, genome of majority of plant species might have undergone one or more of following type of duplications: (1) ancient genome duplication, that occurred >100 MYA (Pfeil et al., 2005), (2) segmental duplication, that contains duplication of several genes in a stretch, (3) tandem duplication that occurs at gene level, and (4) recent genome duplication. However, peaks could be observed only in the case of genome duplication. In past, Ks peaks were compared and analyzed in detail to explain evolutionary processes (Koch et al., 2000; Blanc and Wolfe, 2004; Shoemaker et al., 2006).

**Figure 3:**
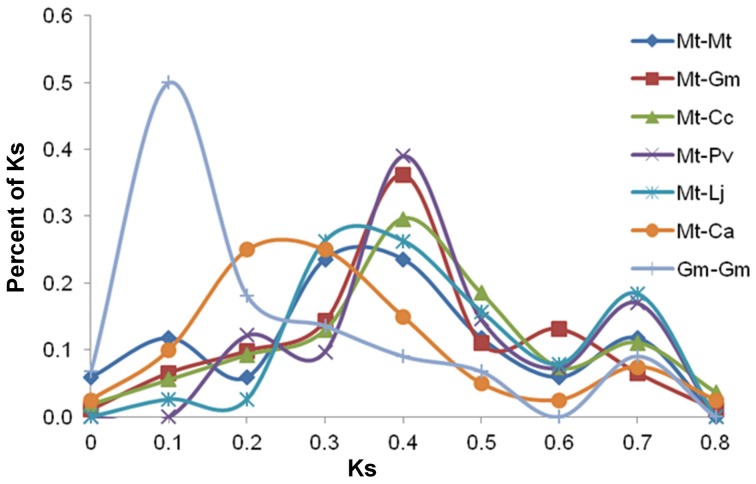
**Ks proportional plot based on sequence data for 20 NF-related genes**. This plot shows peaks based on Ks values between *Mt* and other five legume species and one peak based on Ks values of inparalogs of *Gm* (*Gm*-*Gm*). The plots shows three types of peaks: (i) the first type of peak observed at Ks 0.1 indicates recent genome duplication in *Gm*, (ii) the second type of peaks observed at or near Ks 0.4 indicate speciation, and (iii) the third type of peaks observed at Ks 0.6 or Ks 0.7 correspond to ancient genome duplication.

Comparison of all 20 NF-related genes with their orthologs of all species provided three types of the peaks (Figure 3). The first type of peak was at Ks 0.1 and restricted to only *Gm*-*Gm*. This peak indicates recent whole genome duplication which occurred only in *Gm* genome (Pfeil et al., 2005; Schmutz et al., 2010) but not in any other legume. The second type of peaks at or near Ks 0.4 were present in *Mt*-*Gm, Mt*-*Cc, Mt*-*Pv, Mt*-*Mt*, and *Mt*-*Lj*. In the case of *Mt*-*Ca* orthologs, the second type of peak was, however, present between Ks 0.2 and 0.3. These analyses indicate that speciation might have happened together in Phaseolids species (*Gm, Pv, Cc*) followed by *Lj*, and then *Mt* and *Ca*. The third type of peaks at Ks 0.6 or Ks 0.7 were present in *Mt*-*Ca, Mt*-*Cc, Mt*-*Lj, Mt*-*Pv*, and *Mt-Gm*. This might correspond with ancient duplication event before speciation (Cannon et al., 2006). As it is not known that how frequently rearrangements had happened after ancient duplication, the peaks observed at Ks 0.6 or 0.7 are smaller as compared to the peaks of second and first type. These analyses indicate that there were many deletions and relocations in genomes after ancient duplication (Pfeil et al., 2005; Kim et al., 2009). The third type of peaks were observed in only few studies earlier because these peaks should have come from outparalogs which could be found abundantly in *Gm* but not enough in other legumes (Blanc and Wolfe, 2004; Shoemaker et al., 2006). However, because of the importance of NF, 10 out of 20 NF-related genes have outparalogs in *Pv, Cc, Ca*, and *Lj*. However, complete conserved outparalogs were not observed across all six legumes for any NF-related genes (Supplementary Figure 1). Only *LYK3* and *N56* genes had outparalogs from four species and other genes had less than three outparalogs.

### Differentiation of inparalogs and outparalogs

Phylogenetic analysis provided two types of phylogenetic trees. Five genes had one cluster (clade) that contained only orthologs and 15 genes had two clusters that contained orthologs and outparalagos. Average Ks for orthologs for all 20 NF-related genes is 0.29 but average Ks in outparalogs for 15 NF-related genes is 0.51. For example, there are three *DMI1* orthologs of *Gm* namely, Glyma12g28860, Glyma16g00500, and Glyma19g45310 (Table 1). Glyma12g28860 and Glyma16g00500 are inparalogs to each other because they are in syntenic region and their sequences are very similar (Ks 0.04). But Glyma19g45310 is outparalog to Glyma12g28860 (Ks 0.34) and Glyma16g00500 (Ks 0.34). Phylogenetic tree indicated that Glyma12g28860 and Glyma16g00500 are in the same cluster and Glyma19g45310 belongs to different cluster (Supplementary Figure 1). Similarly, in the case of phylogenetic tree for *NSP1*, in addition to the main clusters that had the *MtNSP1* and its orthologs for *Ca, Lj, Cc, Pv*, and *Gm* (two inparalogs), there is one extra cluster which has two *Mt* genes, two *Pv* genes and one *Gm* gene (Figure 2B). In these genes, Glyma05g22460 and Glyma07g04430 had Ks 0.685, and Glyma05g22460 and Glyma16g01020 had Ks 0.703. In both of these cases, Ks is higher than average Ks of orthologs (0.29). Furthermore, extra 5 genes in the second cluster have high Ks as compared to the genes present in the first cluster. In the first cluster, Glyma07g04430 and Glyma16g01020 are inparalogs but inparalog of Glyma05g22460 might have been deleted. These analyses indicate that in the case of *Gm*, the gene (*NSP1*) of the common ancestor might have undergone one duplication before speciation, one duplication after speciation and in total there might be four genes. However, one of these four genes might have been deleted after recent genome duplication. As a result, the *Gm* genome has only three *NSP1* genes. In another example of *Mt, MtLYK3* (Medtr5g093450) and its three paralogs (Medtr5g093410, Medtr5g093440, and Medtr5g093730), the phylogenetic tree classifies one paralog (Medtr5g093440) with the *MtLYK3* gene (Medtr5g093450) in one cluster and the remaining two paralogs (Medtr5g093410 and Medtr5g093730) in the other cluster (Table 1, Supplementary Figure 1). Their average Ks for inparalogs (Medtr5g093440-Medtr5g093450 and Medtr5g093410-Medtr5g093730) is 0.21 and for outparalogs is 0.39. These analyses suggest that *MtLYK3* has four copies as a result of ancient duplication and then followed by tandem duplication. It is interesting to note that they are located very closely and they have less Ks than other outparalogs. *MtLYK3* is the only one case which has outparalogs together at very close position. On the other hand, in other cases, closely located genes are inparalogs. These cases include *NRT1* genes in *Lj*, N56 genes in *Lj* and *Gm, ENOD93* genes in *Cc* and *Gm, SUNN* genes in *Mt*.

### Interesting cases

Ks value and Ks peaks have been used for understanding of genome evolution in many studies. In our study with 20 NF-related genes, most of the observed cases corresponded to results or hypothesis of previous studies in legumes (Schlueter et al., 2004, 2007; Pfeil et al., 2005; Shoemaker et al., 2006; Shin et al., 2008; Kim et al., 2009). However, there were at least three cases where we don't have sufficient explanation.

1. The Ks proportional plot showed no peak at Ks 0.4 for *Gm*-*Gm* though *Mt*-*Gm* had a peak at Ks 0.4 (Figure 3). In all earlier studies (*Gm*-*Gm, Mt*-*Gm*) based on whole genome sequences, BAC sequences, ESTs or specific gene families, a peak was observed near Ks 0.4. This peak reflected divergence between Hologalegina and Phaseolids (Schlueter et al., 2004, 2007; Pfeil et al., 2005; Shin et al., 2008; Kim et al., 2009; Schmutz et al., 2010).
2. *MtNFP* and *MtNYR3* encode a same lysin motif receptor kinase and these genes are located “next to each other” in *Mt* (Medtr5g018990 and Medtr5g01900), *Gm* (Glyma11g06740 and Glyma11g06750) and *Ca* (Ca_02086 and Ca_02085) (Supplementary Figure 4). On the other hand, orthologs of *NFP* and *NYR3* were present “very near” (and not “next to each other”) in *Pv, Cc*, and *Lj*. In general, the genes which are present “next to each other” are the cases of inparalogs (tandem duplication). However, this (*MtNFP* and *MtNYR3*) seems to be the only case where two independent genes (they might have been duplicated before speciation) are present next to each other and they have their own orthologs. In *Mt* genome sequences, many local gene duplications were found so if those paralogs were retained, sub- or neo-functionalization could be expected, especially essential genes like NF-related genes (Young et al., 2011).
3. The respective phylogenetic trees for *NFP, NIN*, and *ENOD93* genes seem to have outparalogs but Ks of these genes is less than 0.4 (Supplementary Table 1). The phylogenetic trees for these genes have the same type (two clusters) as the other 12 genes (*DMI1, DMI3, ERN1, FLOT2, LIN, LYK3, LYR3, NSP1, NSP2, SKL1, SUNN*, and *N56*) which have outparalogs. Average of Ks values between outparalogs in these 12 genes were >0.40. Several researches in comparing sequences with expression level or their functions suggested that after duplication there was a bias in which genes were retained or silenced (Shoemaker et al., 2006). And even a specific gene or region could have significantly lower Ks (Koch et al., 2000; Schlueter et al., 2007). These researches are similar with our observation in three genes.

## Summary

Although many comparative genomic studies have been conducted using whole genome sequences, BACs and genes, majority of these studies were restricted to one species or some combination of *Mt, Gm, Lj*, and *Pv*. This is the first study that employs comparative sequence analysis of NF-related genes to understand genome evolution of six legumes which include two model legumes (*Lj* and *Mt*), two commercial legumes (*Pv* and *Gm*) and two “so called” orphan legumes (*Cc* and *Ca*). Sequence distances and Ks values suggested that *Gm*-*Cc* is the closest and *Mt*-*Pv* is the farthest in the divergence of six legumes. Low Ka/Ks of NF-related genes indicated that they were conserved in evolution and NF is a functionally essential trait of legumes. Occurrence of the third type of peak near Ks 0.7 and outparalogs in the case of phylogenetic trees for 15 NF-related genes reconfirmed the ancient duplication. Due to large and small scale of rearrangements of DNA during the course of evolution of the six legume species, observation of three interesting cases (no peak at Ks 0.4 in *Gm*-*Gm*, location of two independent genes next to each other and low Ks values for outparalogs in three genes) could not be fully explained. Though a great amount of sequence information is available for these six legume species, we are still in the process of understanding evolution of genes, genomes, and species.

### Conflict of interest statement

The authors declare that the research was conducted in the absence of any commercial or financial relationships that could be construed as a potential conflict of interest.
