# Supplementary material for: Comparative sequence analysis of nitrogen fixation-related genes in six legumes
Source: Front Plant Sci. 2013 Aug 22;4:300. doi: 10.3389/fpls.2013.00300 (PMC3749373; doi:10.3389/fpls.2013.00300)
Supplement: Supplementary file 1 [file DataSheet1.PDF]

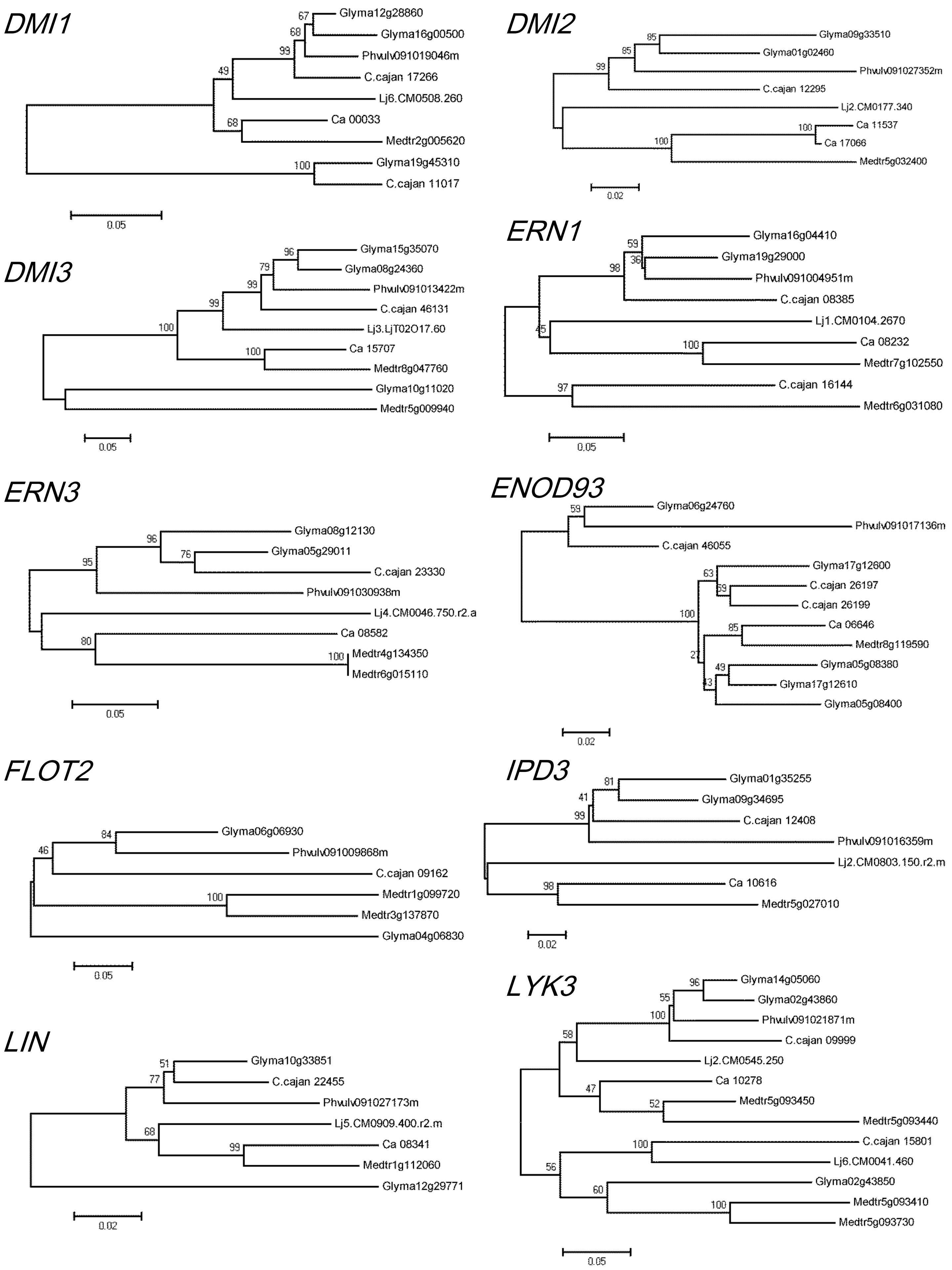
DMI1
Glyma12g28860
Glyma16g00500
Phvulv091019046m
C.cajan 17266
Lj6.CM0508.260
Ca 00033
Medtr2g005620
Glyma19g45310
C.cajan 11017
0.05
DMI2
Glyma09g33510
Glyma01g02460
Phvulv091027352m
C.cajan 12295
Lj2.CM0177.340
Ca 11537
Ca 17066
Medtr5g032400
0.02
DMI3
Glyma15g35070
Glyma08g24360
Phvulv091013422m
C.cajan 46131
Lj3.LjT02O17.60
Ca 15707
Medtr8g047760
Glyma10g11020
Medtr5g009940
0.05
ERN1
Glyma16g04410
Glyma19g29000
Phvulv091004951m
C.cajan 08385
Lj1.CM0104.2670
Ca 08232
Medtr7g102550
C.cajan 16144
Medtr6g031080
0.05
ERN3
Glyma08g12130
Glyma05g29011
C.cajan 23330
Phvulv091030938m
Lj4.CM0046.750.r2.a
Ca 08582
Medtr4g134350
Medtr6g015110
0.05
ENOD93
Glyma06g24760
Phvulv091017136m
C.cajan 46055
Glyma17g12600
C.cajan 26197
C.cajan 26199
Ca 06646
Medtr8g119590
Glyma05g08380
Glyma17g12610
Glyma05g08400
0.02
FLOT2
Glyma06g06930
Phvulv091009868m
C.cajan 09162
Medtr1g099720
Medtr3g137870
Glyma04g06830
0.05
IPD3
Glyma01g35255
Glyma09g34695
C.cajan 12408
Phvulv091016359m
Lj2.CM0803.150.r2.m
Ca 10616
Medtr5g027010
0.02
LIN
Glyma10g33851
C.cajan 22455
Phvulv091027173m
Lj5.CM0909.400.r2.m
Ca 08341
Medtr1g112060
Glyma12g29771
0.02
LYK3
Glyma14g05060
Glyma02g43860
Phvulv091021871m
C.cajan 09999
Lj2.CM0545.250
Ca 10278
Medtr5g093450
Medtr5g093440
C.cajan 15801
Lj6.CM0041.460
Glyma02g43850
Medtr5g093410
Medtr5g093730
0.05

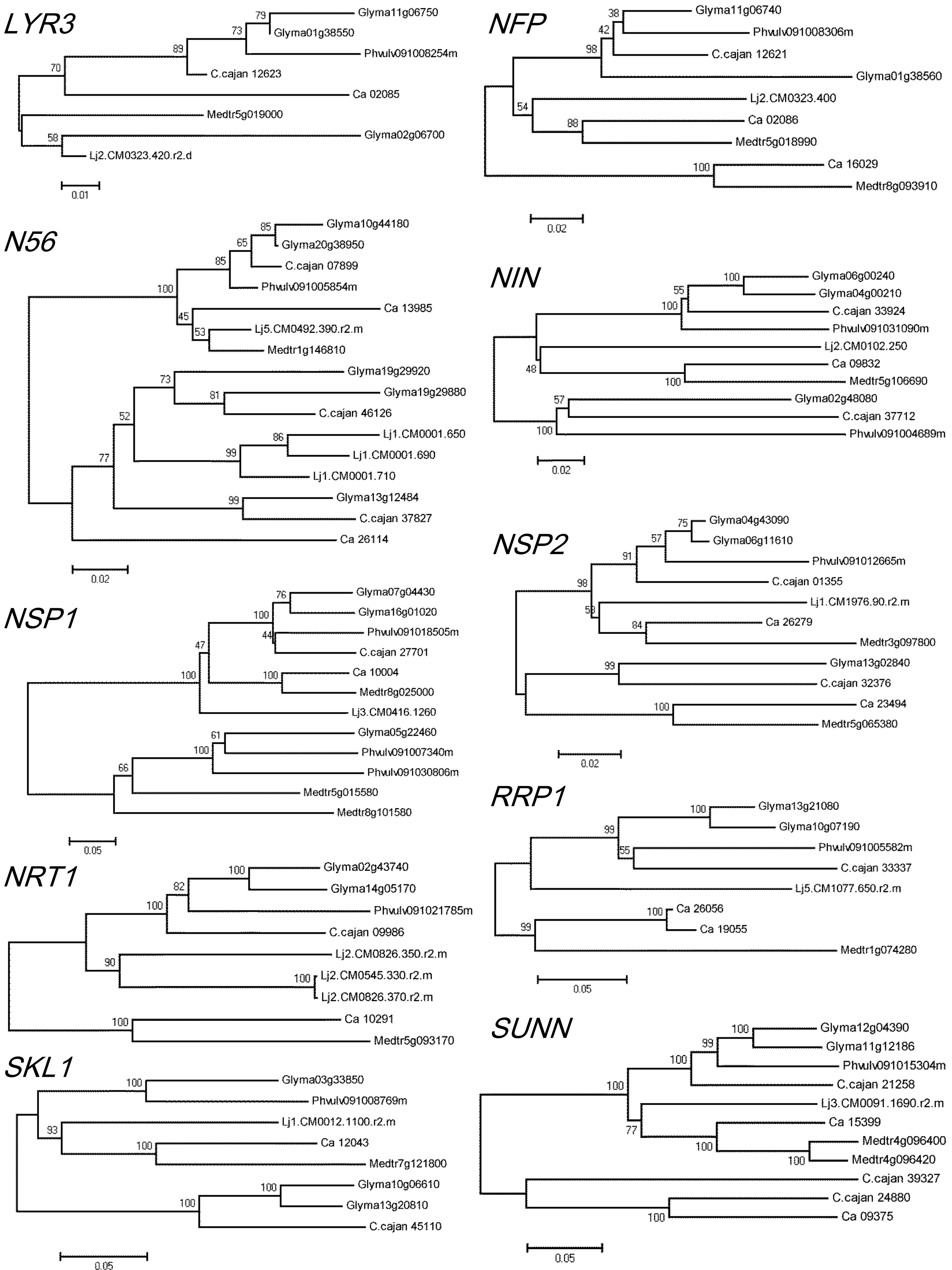

LYR3
Glyma11g06750
Glyma01g38550
Phvulv091008254m
C.cajan 12623
Ca 02085
Medtr5g019000
Glyma02g06700
Lj2.CM0323.420.r2.d
79
73
89
70
58
0.01
NFP
Glyma11g06740
Phvulv091008306m
C.cajan 12621
Glyma01g38560
Lj2.CM0323.400
Ca 02086
Medtr5g018990
Ca 16029
Medtr8g093910
38
42
98
54
88
100
0.02
N56
Glyma10g44180
Glyma20g38950
C.cajan 07899
Phvulv091005854m
Ca 13985
Lj5.CM0492.390.r2.m
Medtr1g146810
Glyma19g29920
Glyma19g29880
C.cajan 46126
Lj1.CM0001.650
Lj1.CM0001.690
Lj1.CM0001.710
Glyma13g12484
C.cajan 37827
Ca 26114
85
65
85
100
45
53
73
81
52
86
99
77
99
0.02
NIN
Glyma06g00240
Glyma04g00210
C.cajan 33924
Phvulv091031090m
Lj2.CM0102.250
Ca 09832
Medtr5g106690
Glyma02g48080
C.cajan 37712
Phvulv091004689m
100
55
100
48
100
57
100
0.02
NSP1
Glyma07g04430
Glyma16g01020
Phvulv091018505m
C.cajan 27701
Ca 10004
Medtr8g025000
Lj3.CM0416.1260
Glyma05g22460
Phvulv091007340m
Phvulv091030806m
Medtr5g015580
Medtr8g101580
76
100
44
47
100
100
61
100
66
0.05
NSP2
Glyma04g43090
Glyma06g11610
Phvulv091012665m
C.cajan 01355
Lj1.CM1976.90.r2.m
Ca 26279
Medtr3g097800
Glyma13g02840
C.cajan 32376
Ca 23494
Medtr5g065380
75
57
91
98
58
84
99
100
0.02
NRT1
Glyma02g43740
Glyma14g05170
Phvulv091021785m
C.cajan 09986
Lj2.CM0826.350.r2.m
Lj2.CM0545.330.r2.m
Lj2.CM0826.370.r2.m
Ca 10291
Medtr5g093170
100
82
100
90
100
100
RRP1
Glyma13g21080
Glyma10g07190
Phvulv091005582m
C.cajan 33337
Lj5.CM1077.650.r2.m
Ca 26056
Ca 19055
Medtr1g074280
100
99
55
100
99
0.05
SKL1
Glyma03g33850
Phvulv091008769m
Lj1.CM0012.1100.r2.m
Ca 12043
Medtr7g121800
Glyma10g06610
Glyma13g20810
C.cajan 45110
100
93
100
100
100
0.05
SUNN
Glyma12g04390
Glyma11g12186
Phvulv091015304m
C.cajan 21258
Lj3.CM0091.1690.r2.m
Ca 15399
Medtr4g096400
Medtr4g096420
C.cajan 39327
C.cajan 24880
Ca 09375
100
99
100
100
77
100
100
100
0.05

Supplementary Figure 1. Phylogenetic trees based on sequences for 20 NF-related genes using neighbor-joining method.
