# Supplementary material for: Comparative sequence analysis of nitrogen fixation-related genes in six legumes
Source: Front Plant Sci. 2013 Aug 22;4:300. doi: 10.3389/fpls.2013.00300 (PMC3749373; doi:10.3389/fpls.2013.00300)
Supplement: Supplementary file 3 [file DataSheet3.PDF]

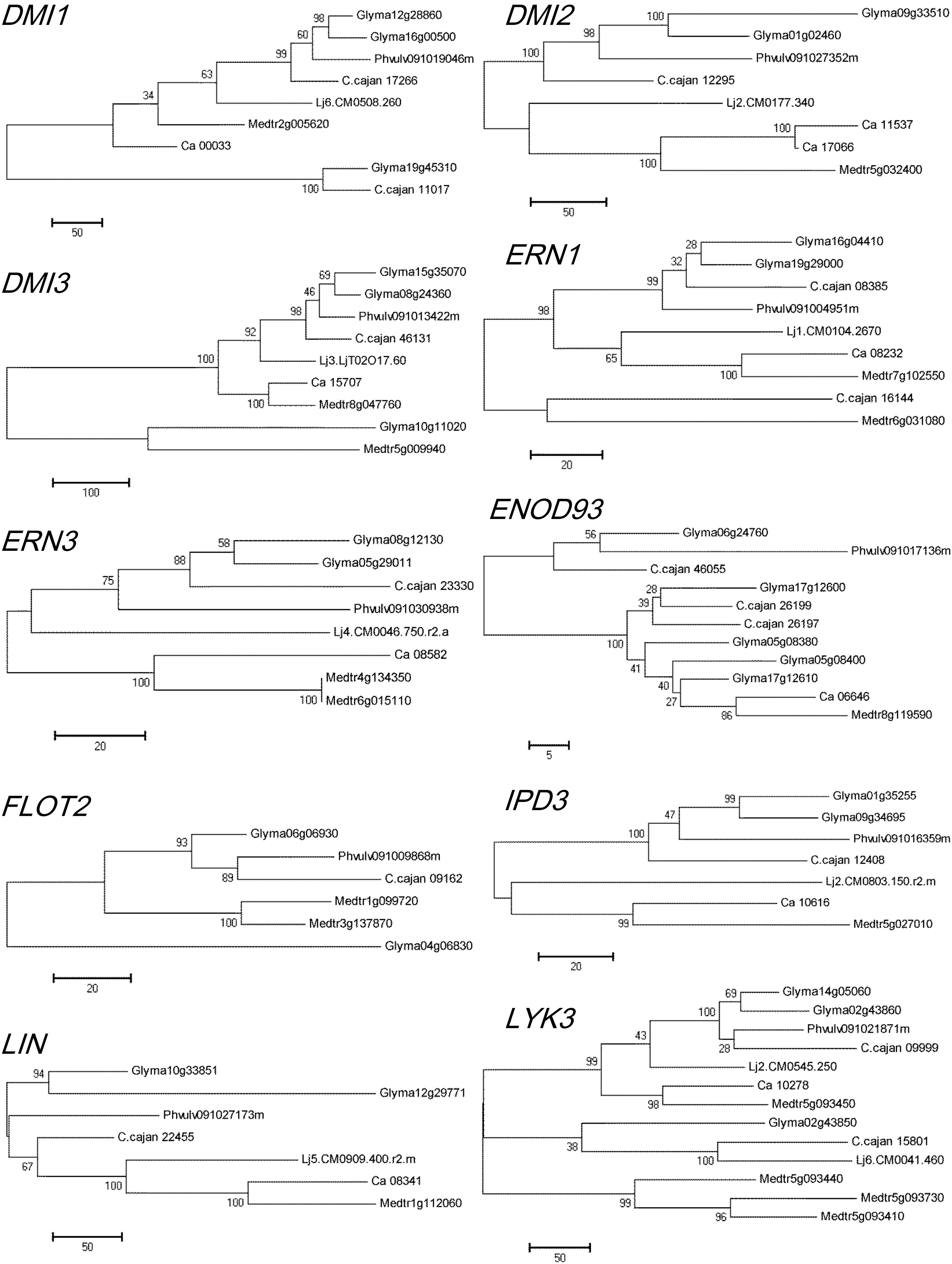
DMI1
98
Glyma12g28860
Glyma16g00500
60
99
Phvulv091019046m
63
C.cajan 17266
34
Lj6.CM0508.260
Medtr2g005620
Ca 00033
Glyma19g45310
100
C.cajan 11017
50
DMI2
100
Glyma09g33510
98
Glyma01g02460
100
Phvulv091027352m
C.cajan 12295
Lj2.CM0177.340
100
Ca 11537
Ca 17066
100
Medtr5g032400
50
DMI3
69
Glyma15g35070
46
Glyma08g24360
98
Phvulv091013422m
92
C.cajan 46131
100
Lj3.LjT02O17.60
Ca 15707
100
Medtr8g047760
Glyma10g11020
Medtr5g009940
100
ERN1
28
Glyma16g04410
32
Glyma19g29000
99
C.cajan 08385
98
Phvulv091004951m
Lj1.CM0104.2670
65
Ca 08232
100
Medtr7g102550
C.cajan 16144
Medtr6g031080
20
ERN3
58
Glyma08g12130
88
Glyma05g29011
75
C.cajan 23330
Phvulv091030938m
Lj4.CM0046.750.r2.a
Ca 08582
100
Medtr4g134350
100
Medtr6g015110
20
ENOD93
56
Glyma06g24760
Phvulv091017136m
C.cajan 46055
28
Glyma17g12600
39
C.cajan 26199
C.cajan 26197
100
Glyma05g08380
41
Glyma05g08400
40
Glyma17g12610
27
Ca 06646
86
Medtr8g119590
5
FLOT2
93
Glyma06g06930
Phvulv091009868m
89
C.cajan 09162
Medtr1g099720
100
Medtr3g137870
Glyma04g06830
20
IPD3
99
Glyma01g35255
47
Glyma09g34695
100
Phvulv091016359m
C.cajan 12408
Lj2.CM0803.150.r2.m
Ca 10616
99
Medtr5g027010
20
LIN
94
Glyma10g33851
Glyma12g29771
Phvulv091027173m
C.cajan 22455
67
Lj5.CM0909.400.r2.m
100
Ca 08341
100
Medtr1g112060
50
LYK3
69
Glyma14g05060
100
Glyma02g43860
43
Phvulv091021871m
28
C.cajan 09999
99
Lj2.CM0545.250
Ca 10278
98
Medtr5g093450
Glyma02g43850
38
C.cajan 15801
100
Lj6.CM0041.460
Medtr5g093440
99
Medtr5g093730
96
Medtr5g093410
50

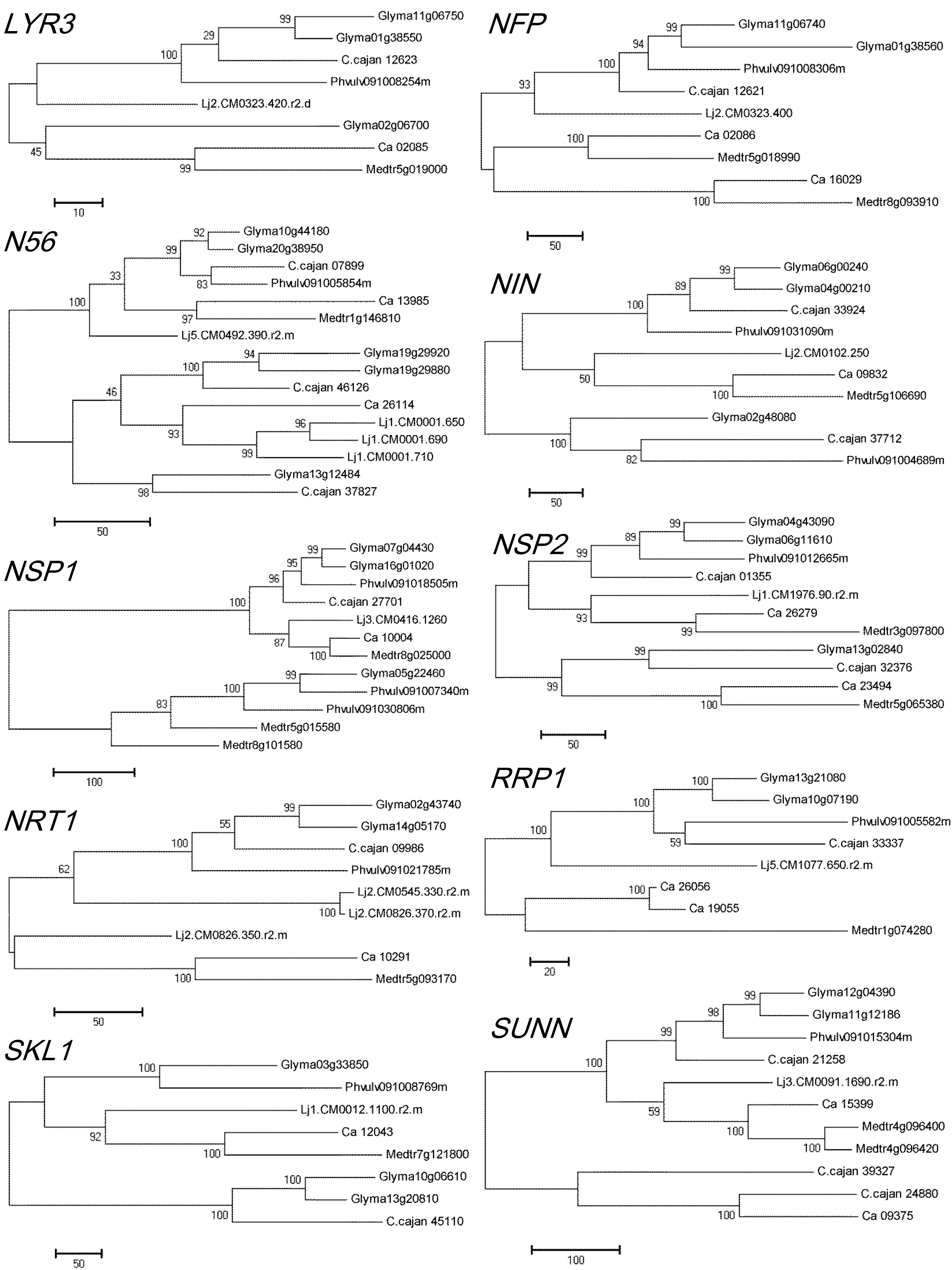
LYR3
Glyma11g06750
Glyma01g38550
C.cajan 12623
Phvulv091008254m
Lj2.CM0323.420.r2.d
Glyma02g06700
Ca 02085
Medtr5g019000
10
NFP
Glyma11g06740
Glyma01g38560
Phvulv091008306m
C.cajan 12621
Lj2.CM0323.400
Ca 02086
Medtr5g018990
Ca 16029
Medtr8g093910
50
N56
Glyma10g44180
Glyma20g38950
C.cajan 07899
Phvulv091005854m
Ca 13985
Medtr1g146810
Lj5.CM0492.390.r2.m
Glyma19g29920
Glyma19g29880
C.cajan 46126
Ca 26114
Lj1.CM0001.650
Lj1.CM0001.690
Lj1.CM0001.710
Glyma13g12484
C.cajan 37827
50
NIN
Glyma06g00240
Glyma04g00210
C.cajan 33924
Phvulv091031090m
Lj2.CM0102.250
Ca 09832
Medtr5g106690
Glyma02g48080
C.cajan 37712
Phvulv091004689m
50
NSP1
Glyma07g04430
Glyma16g01020
Phvulv091018505m
C.cajan 27701
Lj3.CM0416.1260
Ca 10004
Medtr8g025000
Glyma05g22460
Phvulv091007340m
Phvulv091030806m
Medtr5g015580
Medtr8g101580
100
NSP2
Glyma04g43090
Glyma06g11610
Phvulv091012665m
C.cajan 01355
Lj1.CM1976.90.r2.m
Ca 26279
Medtr3g097800
Glyma13g02840
C.cajan 32376
Ca 23494
Medtr5g065380
50
NRT1
Glyma02g43740
Glyma14g05170
C.cajan 09986
Phvulv091021785m
Lj2.CM0545.330.r2.m
Lj2.CM0826.370.r2.m
Lj2.CM0826.350.r2.m
Ca 10291
Medtr5g093170
50
RRP1
Glyma13g21080
Glyma10g07190
Phvulv091005582m
C.cajan 33337
Lj5.CM1077.650.r2.m
Ca 26056
Ca 19055
Medtr1g074280
20
SKL1
Glyma03g33850
Phvulv091008769m
Lj1.CM0012.1100.r2.m
Ca 12043
Medtr7g121800
Glyma10g06610
Glyma13g20810
C.cajan 45110
50
SUNN
Glyma12g04390
Glyma11g12186
Phvulv091015304m
C.cajan 21258
Lj3.CM0091.1690.r2.m
Ca 15399
Medtr4g096400
Medtr4g096420
C.cajan 39327
C.cajan 24880
Ca 09375
100

Supplementary Figure 3. Phylogenetic trees based on sequences for 20 NF-related genes using maximum-parsimony method.
