# Supplementary material for: Comparative sequence analysis of nitrogen fixation-related genes in six legumes
Source: Front Plant Sci. 2013 Aug 22;4:300. doi: 10.3389/fpls.2013.00300 (PMC3749373; doi:10.3389/fpls.2013.00300)
Supplement: Supplementary file 4 [file DataSheet4.PDF]

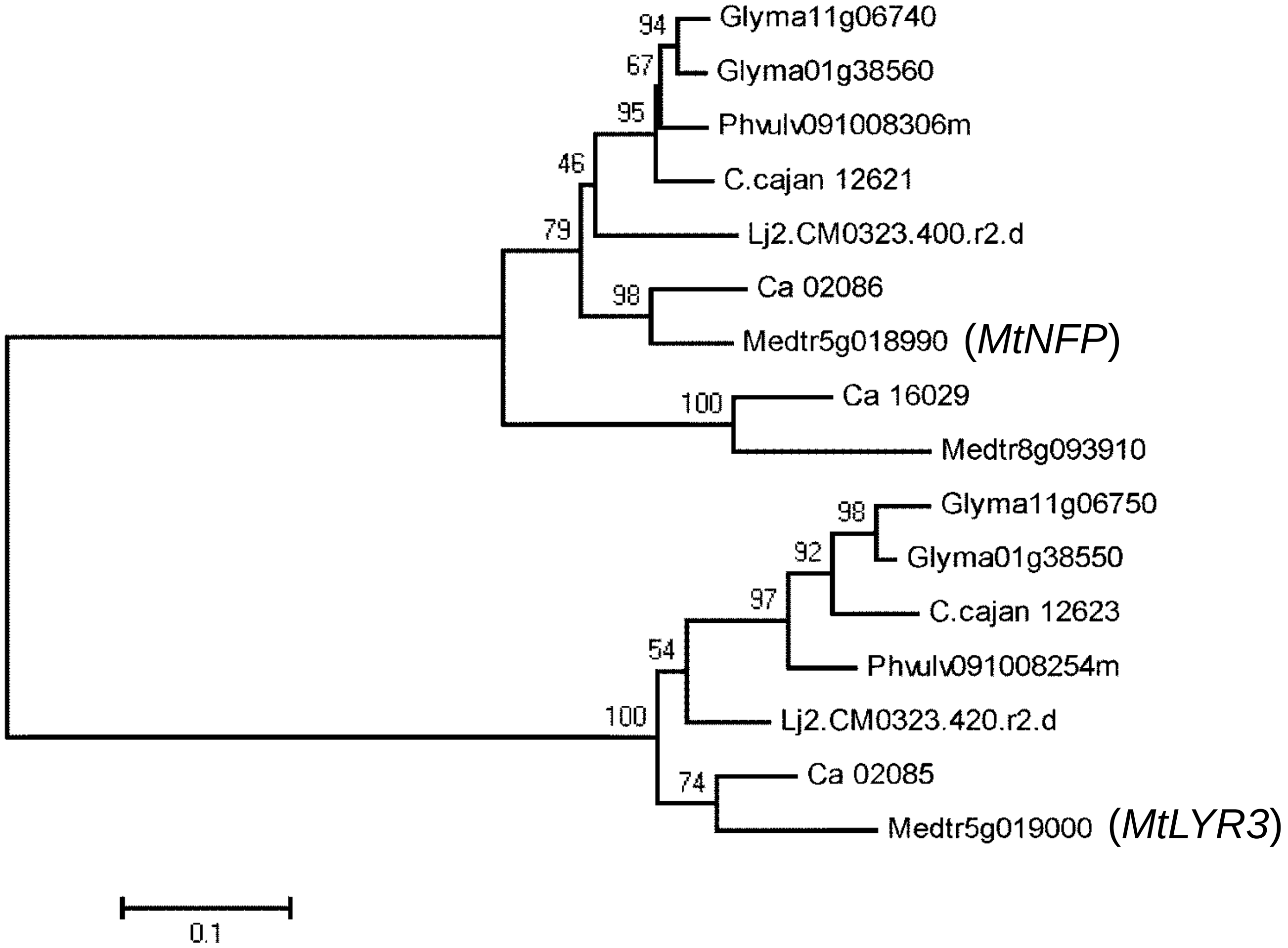

Supplementary Figure 4. Phylogenetic tree based on sequences for lysin motif receptor kinase genes (*NFP* and *LYR3*) genes in six legumes.
